# Draft Genome Sequence of Lactiplantibacillus plantarum NMZ-1139, Isolated from Whisky Mash

**DOI:** 10.1128/MRA.01008-21

**Published:** 2021-12-09

**Authors:** Hiroshi Takagi, Ryota Moriuchi, Yu Kanesaki, Satoshi Katsuyama, Masahiro Suzuki, Reo Mochizuki, Ken Yokozawa, Kenji Iwahara, Hideo Dohra

**Affiliations:** a Numazu Technical Support Center, Industrial Research Institute of Shizuoka Prefecture, Shizuoka, Japan; b Research Institute of Green Science and Technology, Shizuoka University, Shizuoka, Japan; c Graduate School of Integrated Science and Technology, Shizuoka University, Shizuoka, Japan; University of Maryland School of Medicine

## Abstract

Lactiplantibacillus plantarum NMZ-1139 was isolated from whisky mash and applied to sour beer production. Here, we report the draft genome sequence of L. plantarum NMZ-1139, which contains 3,117 protein-coding sequences, including genes associated with hop resistance, such as *horA* and *hitA*.

## ANNOUNCEMENT

Sour beer is a strong acid beer, such as Belgian Lambic, Flanders red ale, Flanders brown ale, German Berliner Weisse, and Gose (1, 2). Traditional sour beers are produced by spontaneous fermentations involving numerous yeast and bacterial species. Therefore, traditional sour beer productions have problems such as inconsistent product quality and long fermentation times. An effective way to solve these problems is to use a single strain of lactic acid bacteria for lactic acid fermentation (3). In this study, we determined the draft genome sequence of Lactiplantibacillus plantarum NMZ-1139, which can be applied to sour beer production.

L. plantarum NMZ-1139 was isolated from whisky mash using de Man-Rogosa-Sharpe (MRS) agar medium and was screened as a lactic acid bacterium that produces sufficient lactic acid in Spraymalt medium (Muntons plc, Suffolk, UK). A single colony of L. plantarum NMZ-1139 was inoculated into MRS broth and incubated at 30°C for 48 h. The genomic DNA was extracted using the NucleoSpin tissue kit (Macherey-Nagel, Germany) and fragmented using an M220 focused ultrasonicator (Covaris Inc., Woburn, MA, USA) with a protocol for 550-bp fragments. A library was prepared using a TruSeq DNA PCR-free library preparation kit (Illumina, San Diego, CA, USA), followed by 301-bp paired-end sequencing on the Illumina MiSeq platform. The raw read sequences were cleaned using Trimmomatic v. 0.39 (4), by trimming adapter sequences and low-quality ends (quality score, <15), and removing reads of less than 150 bp. The resulting 962,210 high-quality read pairs (totaling 536.8 Mb) were assembled using SPAdes v. 3.15.2 (5) with a default set of *k*-mer sizes and options (careful, only-assembler, and cov-cutoff auto). The draft genome consisted of 38 contigs (*N*_50_, 286,078 bp), including one circular plasmid (designated pLP2K), with a total length of 3,335,484 bp and a G+C content of 44.3%. Gene prediction and annotation were performed with DFAST-core v. 1.2.11 (6) by running GeneMarkS2 v. 1.14_1.25 (7), RNAmmer v. 1.2 (8), and tRNAscan-SE v. 2.0.5 (9) for protein-coding sequences (CDSs), rRNA genes, and tRNA genes, respectively. Annotation for CDSs was performed by BLASTp using an in-house database containing 563 genome sequences of L. plantarum in the NCBI RefSeq database as of 26 July 2021. Default parameters were used for all software unless otherwise specified. The genome contains 3,117 CDSs, 5 rRNA genes, and 65 tRNA genes.

Lactobacilli are generally inhibited from growing in beer by the presence of iso-alpha acids, bitter compounds derived from hops. However, some strains containing the genes associated with hop resistance, such as *horA*, *horC*, and *hitA*, can survive in beer (2, 10–12). The draft genome sequence of L. plantarum NMZ-1139 contains genes similar to *horA* and *hitA* (Table 1). The presence of these genes may be advantageous for the production of sour beer. The genomic information for L. plantarum NMZ-1139 will be useful for understanding the mechanisms of its fermentation.

**TABLE 1:** Genes homologous to hop resistance genes in Lactiplantibacillus plantarum NMZ-1139

Hop resistance gene (accession no.) and locus tag	Putative function	Amino acid identity (%)^*a*^	Reference
*horA* (BAA21552.1)			11
NMZ1139_13600	ABC transporter ATP-binding protein	59 (337/568)	
NMZ1139_24780	ABC transporter ATP-binding protein	37 (210/575)	
*hitA* (Q93V04.1)			12
NMZ1139_17100	NRAMP family divalent metal transporter	76 (354/463)	
NMZ1139_13810	NRAMP family divalent metal transporter	55 (250/456)	
NMZ1139_31010	NRAMP family divalent metal transporter	45 (196/433)	

a Percentage of animo acid identities to BAA21552.1 or Q93V04.1 were calculated using blastp. The numbers in parentheses indicate identical amino acids/alignment length of amino acid sequences .

### Data availability.

The raw reads have been deposited in the DDBJ Sequence Read Archive (DRA) under the accession number DRR316760. This whole-genome shotgun sequencing project has been deposited in DDBJ/ENA/GenBank under the accession numbers BPVY00000000 (37 contigs) and AP025175 (plasmid pLP2K).
